# Intrathecal administration of nusinersen for spinal muscular atrophy: report of three cases with severe spinal deformity

**DOI:** 10.1186/s40981-020-00334-7

**Published:** 2020-04-20

**Authors:** Sayo Nakao, Shinichi Yamada, Katsuya Tsuda, Taishi Yokomizo, Teruyuki Sato, Shuichi Tanoue, Teruyuki Hiraki

**Affiliations:** 1 Department of Anesthesiology, Kurume University School of Medicine, 67 Asahi-machi, Kurume, Fukuoka, 830-0011 Japan; 2 Department of Radiology, Kurume University School of Medicine, 67 Asahi-machi, Kurume, Fukuoka, 830-0011 Japan

**Keywords:** Spinal muscular atrophy, Intrathecal administration, Nusinersen, Severe scoliosis

## Abstract

**Background:**

Spinal muscular atrophy (SMA) is a genetic disease characterized by degeneration of the spinal cord, resulting in progressive muscle atrophy. Recently, nusinersen has been approved for treating SMA, which should be administered intrathecally.

**Case presentation:**

Patient 1 was a 36-year-old woman with SMA type 2. Patients 2 and 3 were 10- and 17-year-old girls with SMA type 1. In patients 1 and 2, the needle was inserted into the spinal column, but outflow of cerebrospinal fluid was unable to be confirmed. CT revealed that the dural sac terminated at the L5 level in patients 1 and 3 and at the L5/S1 level in patient 2.

**Conclusions:**

Patients with SMA often present with high-grade scoliosis, making intrathecal administration difficult. In addition, the dural sac may terminate at a level higher than normal. To ensure intrathecal administration, the level of dural sac termination must be confirmed by CT before puncture.

## Background

Spinal muscular atrophy (SMA) is a genetic disorder characterized by degeneration of the anterior horn cells in the spinal cord, resulting in progressive muscle weakness and atrophy [1]. Nusinersen is an antisense oligonucleotide designed to increase the expression of the SMA and is the first appeared drug to treat SMA in the USA and in Japan in 2016 and 2017, respectively. Antisense oligonucleotides do not readily cross the blood-brain barrier, and nusinersen should be administered directly into the cerebrospinal fluid [2]. However, lumbar puncture is often technically challenging in patients with SMA because of young age, spinal deformity, scoliosis, and abnormal spinal rotation during growth. We report three patients with SMA in whom intrathecal administration of nusinersen was difficult because of the abnormal level of the bottom of the dural sac.

## Case presentation

### Case 1

Patient 1 was a 36-year-old woman (130 cm, 18 kg) with a diagnosis of SMA type 2 at the age of 1 year and 3 months. Almost all activities of daily living (ADLs) were performed with assistance, and the patient spent most of the time lying in bed because of high-grade scoliosis and joint contracture. The patient was referred to our department for treatment with intrathecal nusinersen from the pediatric department because of its difficulty under fluoroscopy.

**Fig. 1:**
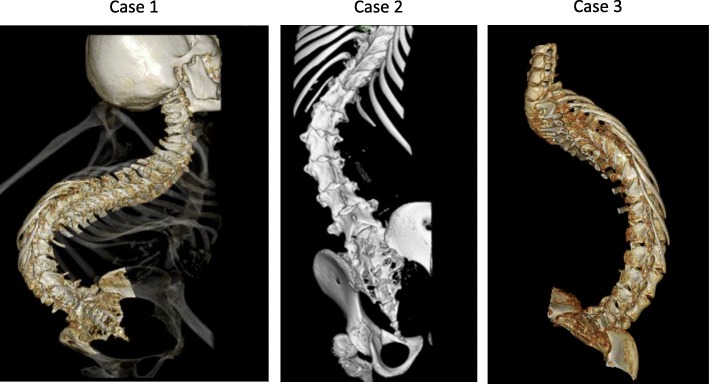
Reconstructed three-dimensional CT images of three cases. The volume-rendering reconstructions show marked scoliosis

Three-dimensional computed tomography (3D-CT) demonstrated that lumbar puncture at the L4/5 and L5/S1 interspace was possible in the prone position (Fig. 1). After a simulation in the interventional radiology suit, the patient was placed in the prone position with the right side slightly elevated on a surgical table, which was adjusted in a 30-degree reverse Trendelenburg position, and the left side was tilted at 45° in order to insert the needle (TOP spinal needle 25 G × 89 mm Quincke, TOP corp., Tokyo, Japan) vertically downward to the intrathecal space in a hybrid operating room.

**Fig. 2:**
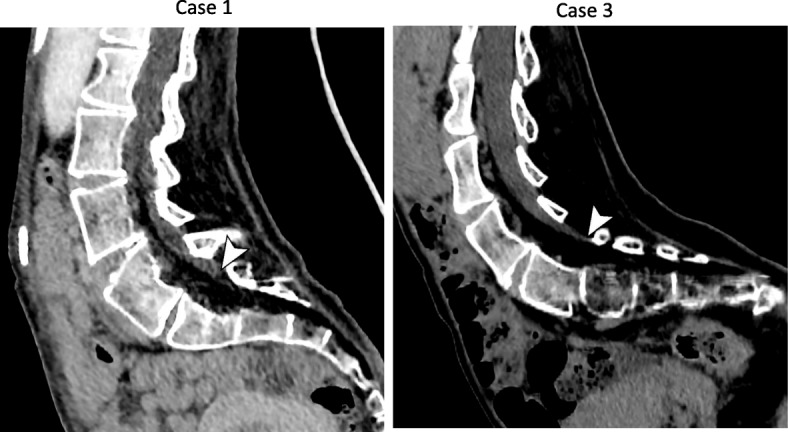
Curved multiplanar reconstructed images in 2 cases (cases 1 and 3). Arrowheads indicate the lower end of the dural sac

Cerebrospinal fluid outflow was not detected after inserting the needle from the L4/5 or L5/S1 interspaces (depth 70 mm), despite the location of the tip of the needle in the spinal column on the fluoroscopic image. Further, examination of CT revealed that the dural sac terminated at the L5 level (Fig. 2). Although the L3/4 was in a difficult position to confirm the laminar spaces on CT, the needle was inserted there (depth 70 mm), cerebrospinal fluid (CSF) outflow was confirmed, and nusinersen 12 mg (5 mL) was administered intrathecally after collection of 5 mL of CSF.

### Case 2

Patient 2 was a 10-year-old girl (135 cm, 18 kg) with a diagnosis of SMA type 1 at the age of 6 months. Tracheostomy was performed when she was 4 years old. ADLs were performed with total assistance; muscle atrophy was significant; and joint contracture was observed. The patient had high-grade scoliosis, making it difficult to select a position. Three-Dimensional CT demonstrated that lumbar puncture at the L4/5 interspace was possible in the lateral decubitus position (Fig. 1).

As the patient was only 10 years old and highly fearful of puncture, propofol (5 mg/kg/h) was administered for sedation. The laminal space was examined under fluoroscopy in the hybrid operating room, and the needle (23 G × 70 mm) was inserted into the L4/5 intervertebral space (depth 30 mm). C-arm CT performed after the puncture confirmed that the tip of the needle was in the spinal column; however, there was no outflow of CSF. Additional CT images confirmed that the dural sac terminated at the level of L5/S1 intervertebral space. The needle was then inserted into the L3/4 (depth 30 mm); CSF outflow was confirmed; and nusinersen 12 mg (5 mL) was administered intrathecally after collection of 5 mL of CSF.

### Case 3

Patient 3 was a 17-year-old girl (139 cm, 22 kg) with a diagnosis of SMA type 1 at the age of 11 months. Three-dimensional CT demonstrated that the lumbar spine was markedly rotated, and performing puncture was expected to be difficult (Fig. 1). Images obtained before the procedure confirmed that the dural sac terminated at the L5 level (Fig. 2); therefore, the needle (25 G × 70 mm) was inserted into the L3/4 (depth 45 mm), and nusinersen 12 mg (5 mL) was administered intrathecally after collection of 5 mL of CSF. There were improvements in motor function of extremities after second administration in all cases.

## Discussion

SMA is an autosomal recessive neuromuscular disorder characterized by progressive muscle atrophy and weakness caused by the degeneration of motor neurons in the anterior horn of the spinal cord. The incidence is 1 per 10,000 live births, and it is one of the genetic causes of high infant mortality rates [3]. The disease is clinically classified as types 1–4. In approximately 60% of infants with SMA type 1, onset is within the first 6 months of life, and the majority cannot survive more than 2 years without respiratory support or nutritional support. Type 2 accounts for approximately 30% of all cases of SMA. Onset is typically within 7–18 months of age, and most affected patients are able to sit independently but unable to walk independently [4, 5]. Patients with type 3 are able to walk but have gradual progression of weakness so that many patients lose this ability over years of the disease [6]. Patients with type IV typically have onset of weakness in the second or third decade of life. Motor impairment is mild without respiratory or nutritional problems, and patients are able to walk in adult years [3].

Nusinersen must be administered intrathecally, and lumbar punctures are often technically challenging because patients with SMA have spinal deformity and scoliosis, and spine rotation occurs during growth. Many techniques have employed the use of fluoroscopy, ultrasound, and cone-beam CT for successful intrathecal puncture [6–8]. Before the initial procedure in each patient, we assessed the position of laminar spaces on 3D-CT and determined the site and angle of the punctures. If the positioning of the patient at the time of puncture is expected to be difficult, we check the position in which the puncture can be performed by fluoroscopy in advance. The actual procedure is performed in a hybrid operating room where high-definition radiographs and c-arm CT images can be obtained. Other advantages of performing this procedure in an operating room include the ability to prevent infection and to ensure safety when difficulty with airway management or sudden changes associated with procedures and sedation occur.

In our three patients, the tip of the needle was inserted into the spinal column, but outflow of CSF was unable to be confirmed. One of the common causes for the lack of CSF outflow is needle tip placement outside the subarachnoid space [9]. The dural sac terminates at the S3–S4 level during the neonatal period and is considered to shift to the S1–S2 level by the age of 1 year [10]. Cadaver, magnetic resonance imaging, and myelographic studies revealed that the dural sac terminates most often at the S2 level, but this location can vary from the L5/S1 to the S4 levels [11–13]. On CT, the dural sac terminated at the L5 level in 2 of our patients and at the L5/S1 level in 1, which were higher levels than normal, and this may have been the reason for the absence of CSF outflow. In these circumstances, even if the tip of the needle was in the spinal column, the lower end of the dural sac may have shifted and tapered, and the tip of the needle may have been located outside the subarachnoid space.

On administration of nusinersen intrathecally to three patients with SMA, we found that the dural sac terminated at a level higher than normal. CT of the lumbar spine must be performed preoperatively to confirm the level of dural sac termination and to determine the site of puncture. We found no previous reports regarding the level of dural sac termination in patients with SMA, and further study with a larger sample is required.

## Data Availability

The data in this case report are available from the corresponding author upon reasonable request.

## Abbreviations

SMA: Spinal muscular atrophy
ADLs: Activities of daily living
3D-CT: Three-dimensional computed tomography
CSF: Cerebrospinal fluid
